# Acute Efficacy of a Traditional Chinese Medicine for Treatment of Frequent Premature Ventricular Contractions in Patients with Concomitant Sinus Bradycardia: Results from a Double-Blind, Placebo-Controlled, Multicentre, Randomized Clinical Trial

**DOI:** 10.1155/2019/3917282

**Published:** 2019-03-04

**Authors:** Fengxiang Zhang, Jiangang Zou, Hao Yu, Xiaorong Li, Pipin Kojodjojo, Xin Cai, Shu Zhang, Congxin Huang, Kui Hong, Bo Yu, Guangping Li, Suxin Luo, Shenghua Zhou, Yang Zheng, Jie Fan, Xuebin Cao, Guizhou Tao, Guotai Sheng, Zhisheng Bai, Shan Jiang, Xiaolin Liu, Weijuan Gu, Feng Chen, Kejiang Cao

**Affiliations:** ^1^Department of Cardiology, The First Affiliated Hospital of Nanjing Medical University, Nanjing, China; ^2^Department of Statistics of Nanjing Medical University, Nanjing, China; ^3^National University Heart Centre, National University Hospital, Singapore; ^4^Department of Cardiology, The First Affiliated Hospital of Bengbu Medical College, Bengbu, China; ^5^Department of Cardiology, Chinese Academy of Medical Sciences Fuwai Cardiovascular Hospital, Beijing, China; ^6^Department of Cardiology, Renmin Hospital of Wuhan University, Wuhan, China; ^7^Department of Cardiology, The Second Affiliated Hospital of Nanchang University, Nanchang, China; ^8^Department of Cardiology, The First Affiliated Hospital of China Medical University, Shenyang, China; ^9^Department of Cardiology, The Second Hospital of Tianjin Medical University, Tianjin, China; ^10^Department of Cardiology, First Affiliated Hospital of Chongqing Medical University, Chongqing, China; ^11^Department of Cardiology, Xiangya No. 2 Hospital Central South University, Changsha, China; ^12^Department of Cardiology, No. 1 Hospital of Jilin University, Changchun, China; ^13^Department of Cardiology, First People's Hospital of Yunnan Province, Kunming, China; ^14^Department of Cardiology, The 252^nd^ Hospital of Chinese People's Liberation Army, Beijing, China; ^15^Department of Cardiology, First Affiliated of Liaoning Medical University, Shenyang, China; ^16^Department of Cardiology, Jiangxi Provincial People's Hospital, Nanchang, China; ^17^Department of Cardiology, The Department of Cardiology, People's Hospital of Baoji City, Baoji, China; ^18^Department of Cardiology, Jinzhou Central Hospital, Jinzhou, China

## Abstract

Pharmacological antiarrhythmic therapy such as beta-blockers in patients with frequent premature ventricular contractions (PVCs) and concomitant bradycardia is challenging. A traditional Chinese medicine, Shensong Yangxin (SSYX), has been effective in treatment of frequent PVCs and sinus bradycardia (SB) in separate patient cohorts. This double-blind, placebo-controlled, multicentre, randomized clinical trial investigates the acute efficacy of SSYX in reducing PVCs burden in patients with concomitant SB. Patients with symptomatic, frequent PVCs, and SB, defined as mean heart rate (MHR) of 45 to 59 beats per min (bpm), were recruited at 33 medical centres in mainland China and randomly assigned by computer to either SSYX or matching placebo for eight weeks. Patients, investigators, and trial personnel were masked to treatment allocation. Primary endpoints were changes in PVCs burden and MHR as assessed by 24-hour Holter monitoring relative to baseline. Secondary efficacy endpoints were subjective symptom score, ECG, and biochemical parameters. Analysis was based on intention-to-treat principles. 333 patients were randomized, of which 166 received SSYX and 167 placebo. Baseline characteristics did not differ. SSYX reduced PVCs burden by 68.2% (*p* < 0.001) and increased MHR by 10.9% (*p* < 0.001) compared to 32.2% and 4.7%, respectively, in the placebo group. SSYX group experienced greater symptomatic improvement (*p* < 0.001). No differences in reported adverse events were seen (20 versus 23). SSYX is an effective antiarrhythmic therapy for symptomatic, frequent PVCs uniquely suited patients with concomitant SB. Clinical trial number was NCT01750775.

## 1. Introduction

Premature ventricular contractions (PVCs) occur commonly in the general population and are generally considered to be a benign condition, provided patients are asymptomatic and free from structural heart disease [1, 2]. Patients with frequent PVCs however can experience distressing symptoms and in a small minority and develop tachycardia-mediated cardiomyopathy if left untreated [3–6]. Effective suppression of frequent PVCs by pharmacological therapy or catheter ablation can ameliorate the resultant cardiac dysfunction. Based on the most recent international consensus statements, beta-blockers or nondihydropyridine calcium channel antagonists are considered first-line medication for PVCs suppression in symptomatic patients [5]. However, administration of these drugs may be complicated by bradycardia and this complication is particularly relevant since the prevalence of PVCs and bradycardia both increases with advancing age [2].

Sheng Song Yang Xin (SSYX) is based on a traditional Chinese medicine prescription that combines 12 different herbs and has been in use for centuries as a treatment for cardiac ailments (supplementary Table 1) [7]. More recently, the same formulation has been standardized, encapsulated, and approved in 2003 as an antiarrhythmic agent by the State Food and Drug Administration of China (Z20030058). SSYX is used commonly in China on its own or in conjunction with conventional antiarrhythmic agents. In a multicentre randomized clinical study, SSYX reduced PVCs burden in patients with symptomatic, frequent PVCs by 74.2% and 65.8% in patients with and without structural heart disease [8]. In a separate, double-blind, randomized, placebo-controlled trial of patients with symptomatic bradycardia that did not meet conventional criteria for permanent pacing, SSYX increased MHR by 13.3% without any serious adverse events reported [9]. Given these attributes of SSYX, we initiated this study to investigate the clinical efficacy and safety of SSYX for patients with symptomatic frequent PVCs with concomitant bradycardia.

## 2. Patients and Methods

### 2.1. Study Design and Participants

The present study was a double-blind, placebo-controlled, multicentre, randomized clinical trial conducted at 33 medical centres in mainland China. Between 10^th^January 2012 and 19^th^ August 2015, patients fulfilling the inclusion criteria were recruited: aged between 18 to 70 years, experiencing symptoms due to frequent PVCs (defined as more than 1,000 PVCs) and MHR of ≥ 45 but less than 60 beats per minute (bpm) during 24 hours of Holter monitoring. Exclusion criteria were significant sinus node disease defined as either sinus arrest ≥ three seconds or mean sinus rate of less than 45 bpm as measured by 24-hour Holter monitoring, second and complete atrioventricular block, sustained (greater than three seconds) atrial or ventricular arrhythmias, acutely reversible causes of arrhythmias such as endocrine or metabolic disturbances, amiodarone therapy, structural heart disease, acute coronary syndrome, heart failure or left ventricular ejection fraction of less than 50% on transthoracic echocardiography. Written informed consent was obtained from each participant by the principle investigator at each site at least five days before randomization. The trial was approved by the First Affiliated Hospital of Nanjing Medical University Research Ethics Committee and was performed in accordance with the ethical guidelines of the Declaration of Helsinki.

### 2.2. Randomization and Masking

Patients were randomly assigned in a 1:1 ratio to either SSYX or matching placebo, using a telephone interactive voice response system. The randomization sequence was generated independently by computer using random permuted blocks, stratified by PVCs count (between 1000 to 3000, 3001 to 5000, and more than 5000 PVCs over 24 hours) and MHR (MHR of between 45 to 49 bpm, 50 to 54 bpm, and 55 to 59 bpm over 24 hours) and retained by an independent trial statistician. Research participants, personnel, and clinicians were masked to treatment allocation.

### 2.3. Procedures

At the enrollment visit, all patients had their medical history taken using a standardized structured questionnaire and underwent complete physical examination, transthoracic echocardiography, and 12-lead electrocardiography (ECG) after giving informed consent. All antiarrhythmic medications were stopped. Each patient completed a symptom score questionnaire written in Chinese, which rated the subjective severity of seven common arrhythmia-related symptoms (translated English version in supplementary Table 2). Blood samples were taken for testing of serum biochemistry, liver, and renal function tests.

After a run-in period of five days (to allow for washout of any antiarrhythmic agents), 24-hour Holter monitoring was performed for all patients to ensure compliance with inclusion and exclusion criteria and establish pretreatment values. One week after enrollment, patients returned for study entry and were randomized to receive study medication in prefilled bottles containing either oral SSYX or matching placebo capsules (Shijiazhuang Yiling Pharmaceutical Co. Ltd, Shijiazhuang, Hebei Province, China) which were identical in size, weight, color, and taste. Each patient is instructed to take four capsules three times daily for eight weeks.

Clinic follow-ups were performed at four and eight weeks, with physical examination, vital signs, symptom score questionnaires, and 24 hours of Holter recordings repeated during the visit each visit. Furthermore, at the eight-week visit, transthoracic echocardiography, laboratory blood assessments, and 12-lead ECG were also repeated. Pill counts were performed to assess compliance. Participants were specifically asked about adverse events at each visit which were recorded. The primary endpoints were changes in PVCs burden and MHR as determined by 24-hour monitoring between recruitment and final follow-up at eight weeks. Secondary endpoints were changes in symptom score and biochemical, echocardiographic, and electrocardiographic parameters. Reduction in PVCs burden by 50% after eight weeks of treatment was arbitrarily defined as a clinically significant reduction and treatment success.

### 2.4. Statistical Analysis

Based on earlier studies, it was estimated that placebo and SSYX could reduce PVCs burden by 10% and at least 40%, respectively. A sample size of 137 participants in each group would give 90% power to detect such a difference in PVCs burden reduction, assuming a two-tailed *α* of 0.025. Allowing for a 20% dropout rate, our target sample size was increased to 330 participants. Statistical analyses were done with SAS (version 9.2). Parametric data, nonparametric data, and categorical variables were summarized with means and standard deviations, medians, and interquartile ranges and percentages, respectively. Endpoints were analyzed based on the intention-to-treat principle with the last observation carried forward to impute any missing outcomes measures after patients have been randomized. Wilcoxon rank-sum tests and t tests were used to analyze changes in PVCs burden and MHR, respectively. *χ*2 and Wilcoxon rank-sum tests were utilized to compare adverse events and compliance rates between two groups. Prespecified subgroup analyses, with stratification based on age, mean baseline heart rate, and PVCs burden were performed. This trial is registered with ClinicalTrials.gov (NCT 01750775).

## 3. Results

### 3.1. Baseline Characteristics

**Figure 1:**
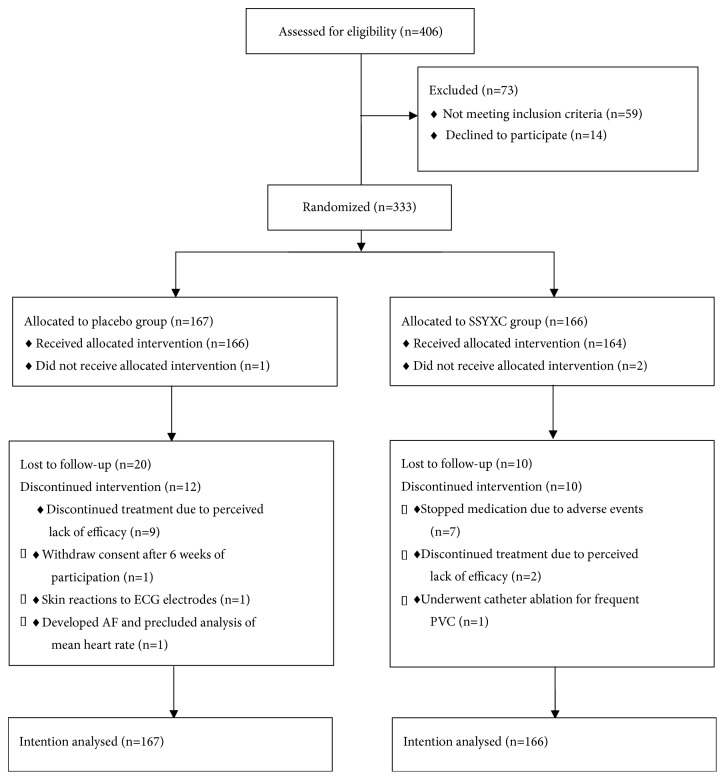
Trial profile with intention-to-treat analysis.

406 patients were assessed for eligibility in this study. Of them, 14 patients declined to participate and 59 patients did not fulfill the inclusion criteria. 333 patients were randomized, of which 166 were assigned to SSYX and 167 to placebo therapy (Figure 1). In the SSYX group, 22 (13.3%) subjects did not complete or violated the study protocol. Two patients did not take SSYX. Ten patients did not attend clinic in accordance to study protocol for follow-up; seven stopped due to adverse clinical events; two had discontinued treatment due to perceived lack of efficacy and one patient underwent curative catheter ablation for frequent PVCs during the study period. In the placebo arm, 33 (19.8%) subjects did not complete or violated the study protocol. One patient did not receive allocated intervention. Twenty patients did not attend clinic in accordance to study protocol for follow-ups; nine had discontinued treatment due to perceived lack of efficacy; one patient withdrew consent after six weeks of participation and one patient developed skin reactions to the adhesive ECG electrodes used for Holter monitoring and withdrew prior to taking medication and a single patient developed persistent AF. However, based on intention-to-treat principles, all patients were included in the final analysis.

Baseline characteristics did not differ between the placebo and the SSYX groups (Table 1). 86.7%of the SSYX group and 80.2% of the placebo group were fully compliant with the treatment protocol.

### 3.2. Primary Endpoints

**Figure 2:**
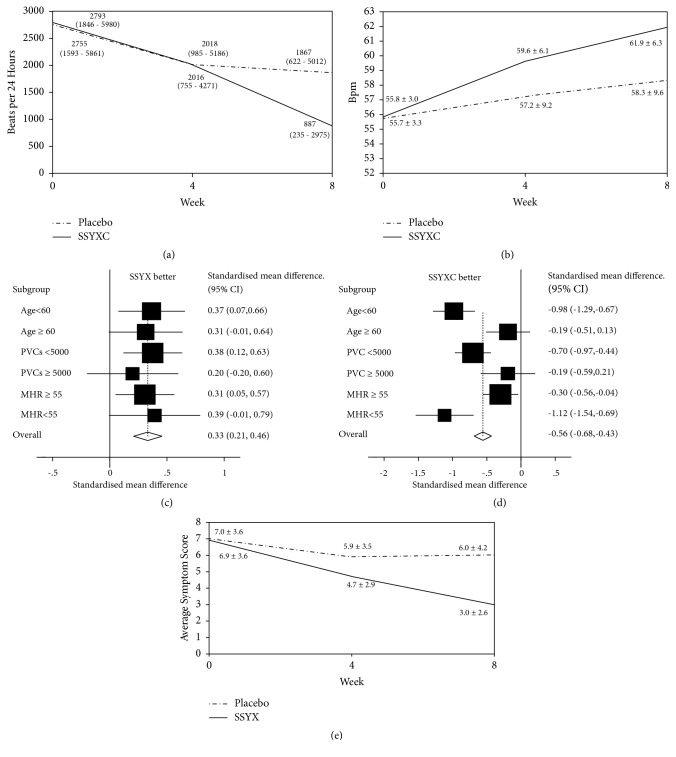
(a) Mean PVC burden during 24-hour Holter monitoring at 0, 4, and 8 weeks in the SSYX and placebo groups. PVC: premature ventricular contraction. (b) Mean heart rates at 0, 4, and 8 weeks between SSYXC and placebo groups. SSYXC: Shensong Yangxin. (c) Subgroup analyses on reduction in PVC burden after 8 weeks of treatment of SSYX stratified according to age, PVC burden, and MHR. MHR: mean heart rate; PVC: premature ventricular contraction; SSYXC: Shen song Yangxin. (d) Subgroup analyses on increase in MHR after 8 weeks of treatment of SSYX stratified according to age, PVC burden, and MHR. MHR: mean heart rate; SSYXC: Shensong Yangxin. (e) Average symptom score 0, 4, and 8 weeks in SSYX and placebo groups. SSYXC: Shensong Yangxin.

Based on intention-to-treat analysis, eight weeks of SSYX therapy significantly reduced PVCs burden compared to placebo. SSYX from 2793 (1846 – 5,980) in 24 hours to 887 (235 – 2,975), 68·2% reduction versus placebo from 2755 (1593 - 5861) to 1867 (622 – 5,012), 32·2% reduction, (p < 0.001) (Figure 2(a)) and 63.3% (105/166) of SSYX and 34.1% (57/167) of placebo patients had a >50% reduction in PVCs burden (p < 0.01). Concurrently, MHR was significantly increased by SSYX compared to placebo (SSYX 55.8 ± 3.0 to 61.9 ± 6.3 bpm, 10.9% increase versus placebo 55.7 ± 3.3 to 58.3 ± 9.6 bpm, and 4.7% increase;* p* < 0.001) (Figure 2(b)).

Prespecified subgroup analysis showed that reduction in PVCs burden was more marked in patients younger than 60 years of age, those with less than 5,000 PVCs per day, and those with a MHR of greater than 55 beats per minute (Figure 2(c)). Younger patients, those with PVCs less than 5,000 per day and those with MHR of less than 55 beats per minute, exhibited a greater increase in heart rate whilst taking SSYX (Figure 2(d)).

### 3.3. Secondary Endpoints

Symptom status in all six domains improved in both SSYX and placebo groups, although the degree of improvement was significantly greater in SSYX treated patients compared to placebo (SSYX 6.9 ± 3.6 to 3.0 ± 2.6 versus placebo 7.0 ± 3.6 to 6.0 ± 4.2;* p *< 0.001) (Figure 2(e)) (supplementary Table 3–5).

Serum biochemistry and renal and liver function tests were unchanged over eight weeks in both groups. ECG parameters including PR, QRS, and QTc intervals remained unchanged over eight weeks (supplementary Table 6).

### 3.4. Safety Analysis

Occurrence of adverse events was similar between the two groups (Table 2). Study participation was terminated in four patients receiving SSYX due to the occurrence of ankle sprain, headache, gastrointestinal discomfort, and new onset of Prinzmetal's angina, respectively. The last adverse event was the only serious adverse event during the conduct of this study. The study medication was stopped and patient's angina symptoms resolved completely with administration of calcium channel blockers. Ten weeks after randomization, all patients who did not complete follow-up were contacted by telephone to confirm that no unreported adverse events or mortality had occurred.

## 4. Discussion

The current, double-blind, multicentre, randomized controlled study demonstrates the effectiveness of a traditional Chinese medicine, SSYX, for the concomitant treatment of frequent PVCs and sinus bradycardia (SB). PVCs burden was significantly reduced by 68.2% whilst MHR increased by 10.9%, resulting in marked symptomatic improvement.

### 4.1. Mechanism of Action of SSYX

Patch-clamping of isolated ventricular myocytes has demonstrated that SSYX is a multichannel blocker with measurable effects on the fast sodium, L-type calcium channel, transient outward potassium (Ito), and inward rectifier potassium currents (IKr), resulting in an overall prolongation of the action potential [10, 11]. Similarly to our findings, sinus rates are increased with SSYX, although this increase is abolished with autonomic blockage with metoprolol and atropine [12]. Whilst attempts have been made with mass spectrometry to identify the most relevant bioactive constituent within SSYX, more than 90 compounds and metabolites are detected and further studies are required to decipher the electrophysiological property of each constituent [13–15].

### 4.2. Clinical Studies of SSYX

Given its long history as an antiarrhythmic formulation in traditional Chinese medicine, SSYX is prescribed commonly in China often in combination with antiarrhythmias, particularly for the treatment of paroxysmal atrial fibrillation (PAF), bradycardia and frequent PVCs. In a meta-analysis of 22 trials involving 2437 PAF patients with treatment courses ranging from eight to 24 weeks, the addition of SSYX to conventional antiarrhythmic agents was found to be beneficial in the maintenance of sinus rhythm and improved quality of care [16]. However, it should be noted that the all trials included in this meta-analysis were deemed by two independent investigators to have a high or unclear risk of bias.

Liu et al. randomized 219 patients from 11 Chinese hospitals with symptomatic bradycardia including those who refused a pacemaker for advanced heart block to either placebo or SSYX. After four weeks of therapy, the MHR, as assessed by 24-hour Holter monitors, increased by 13.3% (mean of 53.4 ± 5.2 bpm to 60.5 ± 8.7 bpm) in the SSYX arm compared to 5.0% (mean of 53.8 ± 4.2 bpm to 56.5 ± 6.0 bpm) in the placebo group [9]. No serious adverse events were reported in this study. Our institution had previously reported parallel double-blind, randomized trials to evaluate the effect of SSYX on frequent PVCs. One hundred and eighty-eight patients with frequent PVCs but without structural heart disease were randomized to receive either eight weeks of SSYX or matching placebo [8]. Use of SSYX was associated with a statistically significant 61.7% reduction in PVCs burden compared to a 17.8 % reduction in the placebo arm. In a parallel arm, 671 patients with frequent PVCs and structural heart disease were randomized to receive either eight weeks of SSYX or mexiletine [8]. PVCs burden decreased by 55.6% in the SSYX group but only 33.5% in the mexiletine group, a difference that was statistically significant. Only five out of 462 patients (1.1%) who received eight weeks of SSYX in these two cohorts experienced minor adverse events such as increased sensation of palpitations, headache, and abdominal discomfort, all of which terminated after discontinuation of treatment.

### 4.3. Unique Clinical Characteristics of SSYX

Antiarrhythmic therapy with beta-blockers and amiodarone are recommended for patients with frequent symptomatic PVCs or resultant tachycardia-mediated cardiomyopathy [17]. However, adverse reactions to such antiarrhythmic drugs include bradycardia and for patients with concomitant bradycardia, the pharmacological options are limited. In a recent study Ling and co-workers randomized 330 patients with frequent right ventricular outflow tract PVCs (averaging more than 13,000 PVCs per day) to either catheter ablation or antiarrhythmic drugs [18]. Of 165 patients assigned to antiarrhythmic drugs, 50 received metoprolol (mean daily dose of 48.2 mg) and 115 received propafenone (mean daily dose of 518.3mg). Approximately 50% of AAD treated patients had a 57% reduction in PVCs burden after one month of therapy. 17 patients (10.3%) experienced drug-related adverse events of which seven (4.2%) patients (three receiving metoprolol and four taking propafenone) was complicated by symptomatic SB.

Therefore, observations from prior clinical trials that SSYX can increase sinus rate and decrease PVCs burden in separate cohorts informed the design and initiation of our current study to validate the clinical effectiveness of SSYX for the treatment of frequent symptomatic PVCs in patients with concomitant bradycardia. Our study findings confirm the unique attributes of SSYX as an unusual antiarrhythmic therapy for frequent PVCs whilst concurrently increasing sinus rates, thus offering greater margin of safety for relatively bradycardic patients. To our knowledge, this is the first randomized trial that specifically investigated antiarrhythmic therapy for patients with symptomatic arrhythmias and concomitant SB.

*Study Limitations. *The duration of administration was short. Although it was sufficient to assess for immediate and short-term adverse reactions, long-term tolerability of SSYX was not assessed. Given the chronicity of medical therapy for common arrhythmias, further long-term studies examining adverse cardiovascular endpoints are required. The administration of four SSYX capsules thrice daily is not conducive for long-term patient compliance. However, due to the multiple complementary components used in most traditional Chinese medicine prescriptions, it is difficult to condense the ingredients into a single pill. Elucidation of the most pertinent bioactive compounds within SSYX may allow for a simpler formulation and improve ease of administration.

## 5. Conclusion

SSYX is an effective and unique antiarrhythmic agent for treatment of frequent symptomatic PVCs and concomitant SB.

## Figures and Tables

**Table 1:** Baseline parameters between SSYXC and placebo group.

	Pla*c*ebo group n = 167	SSYXC group n = 166	p value
*Demographic characteristics*			
Age, years	57.2 (11.3)	56.2 (12.2)	0.45
Male (n)	89 (53%)	76 (46%)	0.17
History of symptoms, years	1.1 (0.5 - 3.3)	1.2 (0.4 - 2.8)	0.59
Height, cm	166.3 (7.6)	165.4 (7.1)	0.30
Weight, kg	65.2 (10.7)	64.4 (10.3)	0.51
*Cardiac parameters*			
SBP, mmHg	126.9 (14.0)	126.8 (13.8)	0.98
DBP, mmHg	78.9 (8.6)	77.4 (8.6)	0.12
LVEF, %	61.7 (6.1)	61.4 (6.0)	0.72
LVDD, mm	48.5 (4.8)	48.6 (4.1)	0.81
Total heart beats per 24 hours	79,510 (8,730)	78,864 (7,764)	0.48
Mean heart rate, bpm	55.8 (3.0)	55.7 (3.3)	0.83
Total PVC, beats per 24 hours	2,755 (1,593 -5861)	2,793 (1,846 -5,980)	0.90
*Symptom score*			
Baseline mean symptom score	7.0 (3.6)	6.9 (3.5)	0.68
*Medical history*			
Hypertension	41 (25%)	50 (30%)	0.25
*Diabetes mellitus*	11 (7%)	12 (7%)	0.82
Hyperlipidemia	3 (2%)	7 (4%)	0.23
*Treatment *			
Aspirin	20 (12%)	22 (13%)	0.73
ACEI/ARB	8 (5%)	9 (5%)	0.79
*Calcium antagonists*	25 (15%)	26 (16%)	0.86
*Diuretics*	4 (2%)	5 (3%)	0.75

SSYX = Shengsong Yangxin, SBP = systolic blood pressure, DBP = diastolic blood pressure, LVDD = left ventricular end-diastolic diameter, LVEF = left ventricular ejection fraction, bpm = beats per min, PVC = premature ventricular contractions, COPD = chronic obstructive pulmonary disease, ACEI = angiotensin converting enzyme inhibitors, and ARB = angiotensin receptor blocker.

**Table 2:** Adverse events reported.

	Placebo group n = 23	SSYX group n = 20
Gastrointestinal disturbance	3	6
Upper respiratory tract infection	3	3
Headache	4	3
Chest discomfort	3	3
Skin rash, itching	1	1
Dry mouth, increased thirst	0	1
*Diarrhea*	1	1
Fever	1	0
Lower respiratory tract infection	1	0
*Diabetes mellitus*	1	0
Exacerbation of chronic obstructive pulmonary disease	1	0
*Duodenal ulcer*	1	0
*Colitis*	1	0
Mouth ulcer	0	1
*Insomnia*	1	0
*Atrial fibrillation*	1	0
Ankle sprain	0	1

SSYX: Shengsong Yangxin.

## Data Availability

The original research data used to support the findings of this study are restricted by Nanjing Medical University Institutional Research Board in order to protect patient privacy. Data may be released from Professor Zhang Feng Xiang (njzfx6@njmu.edu.cn) for researchers who meet the criteria for access to confidential data.

## Supplementary Materials

Supplementary Materials Supplementary Table 1: Constituents of SSYX. SSYXC, Shensong Yangxin. Supplementary Table 2: Standardized symptom severity score (translated from Chinese). Supplementary Table 3: Symptom severity score at baseline. Supplementary Table 4: Symptom severity score after four weeks of treatment. Supplementary Table 5: Symptom severity score after eight weeks of treatment. Supplementary Table 6: Biochemical and ECG parameters at baseline and eight weeks of treatment.
