# Supplementary material for: Acute Efficacy of a Traditional Chinese Medicine for Treatment of Frequent Premature Ventricular Contractions in Patients with Concomitant Sinus Bradycardia: Results from a Double-Blind, Placebo-Controlled, Multicentre, Randomized Clinical Trial
Source: Evid Based Complement Alternat Med. 2019 Mar 4;2019:3917282. doi: 10.1155/2019/3917282 (PMC6425419; doi:10.1155/2019/3917282)
Supplement: Supplementary Materials — Supplementary Table 1: Constituents of SSYX. SSYXC, Shensong Yangxin. Supplementary Table 2: Standardized symptom severity score (translated from Chinese). Supplementary Table 3: Symptom severity score at baseline. Supplementary Table 4: Symptom severity score after four weeks of treatment. Supplementary Table 5: Symptom severity score after eight weeks of treatment. Supplementary Table 6: Biochemical and ECG parameters at baseline and eight weeks of treatment. [file 3917282.f1.docx]

**Supplementary** **tables**

**Supplementary TABLE 1:** Constituents of SSYX. SSYXC, Shensong Yangxin.

**Supplementary TABLE 2:** Standardized symptom severity score (translated from Chinese).

**Supplementary TABLE 3:** Symptom severity score at baseline.

**Supplementary TABLE 4:** Symptom severity score after four weeks of treatment.

**Supplementary TABLE 5:** Symptom severity score after eight weeks of treatment.

**Supplementary TABLE 6:** Biochemical and ECG parameters at baseline and eight weeks of treatment.

**Supplementary TABLE 1: Constituents of Shens Yangxin (SSYX) – a traditional Chinese medical prescription that consists of 12 different traditional Chinese herbs / medical ingredients that have been used since 13^th^ century to improve heart function and regulate normal heart rhythm.**

| **Number** | **Common Name** | **Botanical Name** | **Chinese name** |
| --- | --- | --- | --- |
| 1 | Ginseng | *Ginseng* | 人参 |
| 2 | Dwarf lilyturf | *Ophiopogon* | 麦冬 |
| 3 | Five-flavor berry | *Schisandrachinensis* | 五味子 |
| 4 | Taxillus | *Taxilluschinensis* | 桑寄生 |
| 5 | Macrocarpium Fruit | *Cornus officinalis* | 山萸肉 |
| 6 | Spine Date Seed | *Semen ZiziphiSpinosae* | 酸枣仁 |
| 7 | Red Sage | *Salvia miltiorrhiza* | 丹参 |
| 8 | Red Paeoniae | *PaeoniaeRubra Radix* | 赤芍 |
| 9 | Ground Beetle | *Eupolyphaga* | 土鳖虫 |
| 10 | Chinese Nardostachys | *Nardostachyos Radix et Rhizoma* | 甘松 |
| 11 | Dragon Bones | *Apatite;Calcite* | 龙骨 |
| 12 | Chinese goldthread | *Coptischinensis* | 黄连 |

Shensong Yangxin capsules


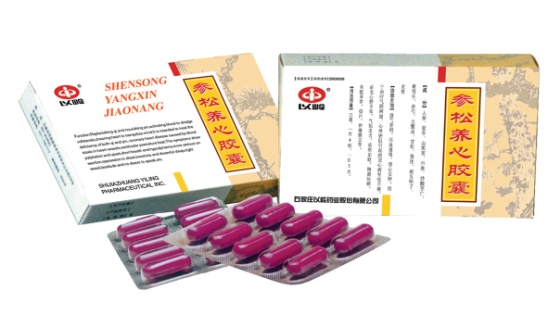


**Supplementary TABLE 2: Symptom severity score questionnaire (a higher score implies more severe symptoms).**

| **Domains** | **Descriptions** | **Score** |
| --- | --- | --- |
| Palpitations | 0 No symptoms  2 Occasional palpitations  4 Frequent palpitations, partially impairing daily activities  6 Frequent palpitations, severely impairing daily activities |  |
| Dyspnoea | 0 No symptoms  1 Occasional dyspnoea without impairment of daily activities  2 Shortness of breath on mild to moderate exertion  3 Shortness of breath at rest |  |
| Fatigue | 0 No symptoms  1 Occasional fatigue but can undertake heavy exertion  2 Fatigue with mild exertion, affecting daily activities  3 Fatigue at rest, affecting daily activities |  |
| Chest pain / tightness | 0 No symptoms  1 Occasional chest pain / tightness, lasting for minutes and relieved with rest  2 Increased frequencies of chest pain / tightness, lasting about at least 10 minutes, relieved with rest  3 Frequent and extended bouts of chest pain / tightness, affecting daily activities and requiring nitroglycerine for relief. |  |
| Insomnia | 0 No symptoms  1 Easy wakening  2 Sleep time more than 4 hours, frequent nightmares, does not affect work  3 Insomnia, palpitations, impairment of daily activities |  |
| Lassitude | 0 No symptoms  1 Lassitude, not talkative, only responds to questions  2 Mental fatigue and day-time sleepiness, occasional response to questions  3 Extreme mental fatigue, occasional words |  |
| Night sweating | 0 No symptoms.  1 Mild night sweating limited to the head area  2 Mild night sweating on chest and back  3 Profuse night sweating of whole body |  |
| **TOTAL SCORE (MINIMUM =0, MAXIMUM 24)** | |  |

**Supplementary TABLE 3: Symptom severity score at baseline.**

| **Symptom** | **Group** | **0** | **1** | **2** | **3** | **P value** |
| --- | --- | --- | --- | --- | --- | --- |
| Shortness of breath | Placebo group (n = 167) | 24 | 109 | 27 | 7 | 0.15 |
|  | SSYXC group (n = 166) | 37 | 98 | 26 | 5 |  |
| Fatigue | Placebo group (n = 167) | 44 | 81 | 38 | 4 | 0.89 |
|  | SSYXC group (n = 166) | 40 | 90 | 32 | 4 |  |
| Chest pain / tightness | Placebo group (n = 167) | 78 | 72 | 16 | 1 | 0.13 |
|  | SSYXC group (n = 166) | 67 | 73 | 23 | 3 |  |
| Insomnia | Placebo group (n = 167) | 65 | 77 | 21 | 4 | 0.81 |
|  | SSYXC group (n = 166) | 64 | 82 | 16 | 4 |  |
| Lassitude | Placebo group (n = 167) | 91 | 64 | 12 | 0 | 0.19 |
|  | SSYXC group (n = 166) | 104 | 49 | 12 | 1 |  |
| Night sweating | Placebo group (n = 167) | 102 | 51 | 13 | 1 | 0.10 |
|  | SSYXC group (n = 166) | 114 | 46 | 6 | 0 |  |
| Overall Score | Placebo group (n = 167) | 404 | 454 | 127 | 17 | 0.25 |
|  | SSYXC group (n =166) | 426 | 438 | 115 | 17 |  |

**Supplementary TABLE 4: Symptom score severity score after four weeks of treatment.**

| **Symptom** | **Group** | **0** | **1** | **2** | **3** | **P value** |
| --- | --- | --- | --- | --- | --- | --- |
| Shortness of breath | Placebo group (n = 167) | 38 | 95 | 33 | 1 | < 0.01 |
|  | SSYXC group (n = 166) | 61 | 94 | 9 | 2 |  |
| Fatigue | Placebo group (n = 167) | 52 | 85 | 26 | 4 | < 0.01 |
|  | SSYXC group (n = 166) | 75 | 79 | 11 | 1 |  |
| Chest pain / tightness | Placebo group (n = 167) | 80 | 72 | 12 | 3 | 0.07 |
|  | SSYXC group (n = 166) | 95 | 61 | 10 | 0 |  |
| Insomnia | Placebo group (n = 167) | 80 | 71 | 14 | 2 | 0.03 |
|  | SSYXC group (n = 166) | 95 | 66 | 5 | 0 |  |
| Lassitude | Placebo group (n = 167) | 113 | 46 | 8 | 0 | 0.31 |
|  | SSYXC group (n = 166) | 122 | 34 | 10 | 0 |  |
| Night sweating | Placebo group (n = 167) | 117 | 44 | 6 | 0 | 0.23 |
|  | SSYXC group (n = 166) | 125 | 40 | 1 | 0 |  |
| Overall Score | Placebo group (n = 167) | 480 | 413 | 99 | 10 | < 0.01 |
|  | SSYXC group (n =166) | 573 | 374 | 46 | 3 |  |

**Supplementary TABLE 5: Symptom score severity score after eight weeks of treatment.**

| **Symptom** | **Group** | **0** | **1** | **2** | **3** | **P value** |
| --- | --- | --- | --- | --- | --- | --- |
| Shortness of breath | Placebo group (n = 167) | 43 | 89 | 29 | 6 | <0.01 |
|  | SSYXC group (n = 166) | 86 | 75 | 4 | 1 |  |
| Fatigue | Placebo group (n = 167) | 59 | 82 | 23 | 3 | <0.01 |
|  | SSYXC group (n = 166) | 101 | 58 | 6 | 1 |  |
| Chest pain / tightness | Placebo group (n = 167) | 79 | 69 | 15 | 4 | <0.01 |
|  | SSYXC group (n = 166) | 116 | 44 | 6 | 0 |  |
| Insomnia | Placebo group (n = 167) | 83 | 69 | 11 | 4 | <0.01 |
|  | SSYXC group (n = 166) | 126 | 38 | 2 | 0 |  |
| Lassitude | Placebo group (n = 167) | 102 | 54 | 10 | 1 | <0.01 |
|  | SSYXC group (n = 166) | 145 | 19 | 2 | 0 |  |
| Night sweating | Placebo group (n = 167) | 113 | 44 | 10 | 0 | <0.01 |
|  | SSYXC group (n = 166) | 150 | 14 | 2 | 0 |  |
| Overall Score | Placebo group (n = 167) | 479 | 407 | 98 | 18 | <0.01 |
|  | SSYXC group (n =166) | 724 | 248 | 22 | 2 |  |

**Supplementary TABLE 6: Biochemical and ECG parameters at baseline and after eight weeks of treatment.**

|  | **Placebo (n=167)** | |  | **SSYX (n = 166)** | |  |
| --- | --- | --- | --- | --- | --- | --- |
| **Biochemical parameters** | Baseline | 8 weeks | **P values** | Baseline | 8 weeks | **P values** |
| **Glutamic-pyruvic transaminase**(IU/L) | 22.0 (11.3) | 23.3 (10.1) | 0.77 | 22.0 (11.4) | 23.1 (10.8) | 0.40 |
| **Glutamic oxalacetic transaminase**(IU/L) | 22.2 (7.1) | 24.3 (7.1) | 0.01 | 22.5 (8.4) | 23.4 (6.7) | 0.30 |
| **Creatinine**(µmol/L) | 70.5 (15.6) | 72.2 (15.5) | 0.15 | 70.3 (16.0) | 69.5 (15.4) | 0.82 |
| **Fasting blood glucose**(µmol/L) | 5.4 (1.2) | 5.5 (0.9) | 0.83 | 54 (0.9) | 5.4 (0.9) | 0.65 |
| **ECG parameters** | | | | | | |
| PR interval (ms) | 161.3 (27.6) | 156.7 (18.7) | 0.77 | 157.4 (27.1) | 162.1 (27.3) | 0.37 |
| QRS duration (ms) | 100.0 (17.4) | 97.5 (15.0) | 0.48 | 102.1 (39.0) | 96.2 (15.3) | 0.36 |
| QT interval (ms) | 424.7 (42.4) | 412.3 (48.2) | 0.60 | 420.1 (53.1) | 419.3 (33.3) | 0.64 |
| QTC (ms) | 418.1 (32.7) | 413.9 (35.5) | 0.77 | 415.7 (50.3) | 422.0 (29.1) | 0.89 |
